# Application of Music Therapy in General Surgical Treatment

**DOI:** 10.1155/2021/6169183

**Published:** 2021-09-28

**Authors:** Jing Liang, Xiaofeng Tian, Wei Yang

**Affiliations:** ^1^Department of Gastrointestinal and Colorectal Surgery, The China-Japan Union Hospital of Jilin University, Jilin, China; ^2^Department of Hepatopancreatobiliary Surgery, The China-Japan Union Hospital of Jilin University, Jilin, China

## Abstract

With the increasing application of music therapy in clinical practice, the effectiveness of music therapy in improving the negative emotions of patients, relieving pain, and adjusting the physiological state has also been receiving increasing recognization. Moreover, music therapy as adjuvant therapy for conventional treatment can achieve a better improvement in patient satisfaction and facilitate the acceptance of make music therapy by the medical industry. In addition to inevitable trauma, general surgery is criticized for its long treatment cycles and postoperative pain. With the continuous development of fast-track surgery (FTS), music therapy has received more attention in general surgical treatment. This study reviews the development history and prospects of music therapy in general surgery.

## 1. Introduction

Department of general surgery is a clinical discipline that treats the liver, biliary tract, pancreas, gastrointestinal, anorectal, vascular diseases, thyroid and breast tumors, trauma, and other diseases with surgical methods, which is the largest department in the surgical system. Psychological and physical pressures are inevitable for patients undergoing general surgery, which compromises the effectiveness of the surgery. Furthermore, postoperative pain, physiological stress reaction, or even postoperative complications promote the occurrence of anxiety, resulting in a vicious circle of “preoperative anxiety - high surgical risk-delayed postoperative recovery - postoperative anxiety - aggregated delayed postoperative recovery and even poor prognosis.” Music therapy (MT) is an emerging discipline that contributes to the elimination of psychological barriers and the restoration or improvement of physical and mental health of the patients through various specially designed musical behaviors, with the theories and methods of psychotherapy as the basis and the unique physical and psychological effects of music as guidance. In short, MT is to improve clinical efficacy and increase palliative benefit with the joint participation of music therapists [1]. Modern medicine has been witnessing a shift from a “biomedicine” model to a “bio-psycho-social medicine” model [2], which encourages the application of MT in increasing clinical practices. This study summarizes the current application of MT in general surgery departments by studying recent domestic and foreign literature, analyzes the limitations of current MT, and proposes future expectations.

## 2. The History of MT

First proposed by the ancient Greek philosopher Pythagoras [3], the concept of MT was then accepted and transformed into a view of “Music Doctor” in 1890 by the Austrian doctor Lichtendal, followed by the first application of MT in surgery [4] in 1914 by the American doctor Evan Kane, who deemed it as an optimal method to “calm down the patients and divert their attention from fear.” In the 1940s, MT was officially recognized as adjuvant therapy in the United States. MT started rather late in China. In 1979, Professor Liu Bangrui, a doctor of MT in the United States, was invited to give lectures at the Central Conservatory of Music. It was the first time that European and American MT were introduced to China, which initiated the construction of MT in China. Thereafter, the establishment of the Chinese Music Therapy Society in 1989 actively promoted the development of MT. However, instead of mere music appreciation, Bruscia defines MT as a systematic intervention process, in which the therapist adopts various forms of music experience and the therapeutic relationship developed during the treatment process as the driving force of the treatment to help the patients achieve the goal of full recovery [5]. After nearly 80 years of systematic development, with its economical and effective features, it has been progressively used in anesthesiology [6], orthopedics [7], pediatrics [8], oncology [9], obstetrics, and gynecology, which has achieved remarkable clinical results. From the aspect of traditional Chinese medicine, the combination of Western MT with the Chinese Five Elements Theory pioneered the development of the “Five Elements MT,” which has commenced to present its advantages with continuous clinical research.

## 3. The Mechanism and Evaluation Criteria of MT

### 3.1. The Mechanism of MT

Currently, the research on the mechanism of MT reveals three possible mechanisms of action: (1) resonance: music is a kind of material energy that affects people physiologically and physically. It transmits information through factors such as tune, rhythm, melody, strength, and speed and regulates periodic physiological movements through resonance response, such as breathing, heartbeat, and blood circulation [10]. Hughes and Fino [11] studied 402 classical music pieces by 59 composers such as Mozart, Patton, and Chopin and concluded that “music may resonate with the structure of the cerebral cortex and may also be related to brain coding.” (2) Theory of the limbic system: dopamine is the most abundant catecholamine neurotransmitter in the brain which regulates various physiological functions of the central nervous system and plays a crucial role in the reward pathway of the brain. MT can stimulate the limbic system of the brain related to emotions, thereby promoting the secretion of endorphins and enhancing the excitability of the parasympathetic nerves to achieve the effect of relaxation [12]. Research by Ferreri et al. [13] found that music, similar to levodopa, can promote the production of dopamine to generate a sense of pleasure in the brain. (3) Brainstem network theory: music can pass in impulses through the ascending projection system of the brainstem network structure, thereby regulating the activity level of the central nervous system and exerting the effect of regulating the psychological and physiological state of the human body [14].

### 3.2. Evaluation Criteria

The effectiveness of MT is evaluated from two aspects: subjective feelings of patients and objective observation by medical staff. First, the patient's subjective feelings include the degree of pain and anxiety. The evaluation of pain degree mainly includes the following six methods: Verbal Description Scale (VDS), Faces Pain Scale-Revised (FPS-R), Verbal Rating Scale (VRS), Visual Analogue Scale (VAS), Numerical Rating Scale (NRS), or Face, Legs, Activity, Crying, Consolability scale (FLACC) [15]. Patient anxiety is basically assessed using the Hospital Anxiety and Depression Scale (HAD), Chinese State-Trait Anxiety Inventory (C-STAI), and Hamilton Anxiety Scale (HAMA). Second, the objective observations of medical staff mainly include various vital signs, such as heart rate (HR), respiratory rate (RR), systolic blood pressure (SBP), diastolic blood pressure (DBP), and oxyhemoglobin saturation (SaO2).

## 4. Application of MT in Preoperative Nursing of General Surgery

The negative emotions such as tension and anxiety in general surgery may lead to abnormal sleep, heart rhythm, and blood pressure, thus, compromising the surgical effect. Research has pointed out a relationship between preoperative anxiety and postoperative acute pain [16]. In addition, the high level of preoperative anxiety increases the incidence of high postoperative pain and the demand for painkillers and anesthetics, leading to delays in recovery and discharge [17]. In traditional clinical methods, the mere use of sleeping pills may trigger corresponding adverse reactions and even drug addiction. In contrast, the consensus of the role of MT in relieving anxiety has been developed in the clinic. The study by Nelson et al. [18] confirmed the effective relieving effect on the anxiety of patients by preoperative MT. Subsequently, Kain et al. [19] further demonstrated that for children, the effect of MT on eliminating anxiety is more obvious with parents' company through the study of 70 cases of preoperative anxiety in children undergoing surgery. The abovementioned studies also showed that MT yields a significant effect on alleviating anxiety, with little impact on patients' heart rate and blood pressure.

## 5. Application of MT in General Surgery

Surgery anxiety is rather frequently observed in patients, especially in those receiving nongeneral anesthesia, as various stimuli during the surgery can be perceptible. In addition, the activation of the sympathetic nervous system may generate different physiological reactions, such as the increase of heart rate, blood sugar level, and blood pressure, and bronchiectasis and peripheral vasoconstriction [20], which will undoubtedly compromise the curative effect of surgery and postoperative outcomes. Jiménez-Jiménez et al. [21] conducted a comparative study of intraoperative MT and nonintraoperative MT on 40 patients and concluded no statistical difference between the control group and the experimental group in the heart rate gradient or the systolic and diastolic blood pressure measured after the intervention. The anxiety state and the pressure perception scale scores after the operation were significantly lower in the MT group (the rates of decreased anxiety levels were 94.7% and 57.9%, *P* < 0.05, and the stress scores were 1.31 and 2.36, *P* < 0.05), which demonstrates that the application of MT during the operation can effectively relieve the pressure and anxiety of patients. A prior study applied the bispectral index (BIS) [22] in a controlled study on 80 patients and found that at 15 minutes after operation, the BIS indexes of the experimental group and the control group were 94.5 and 98.6, respectively (*P* > 0.05), and at 30 minutes they were 79.7 and 98.0, respectively (*P* < 0.05), with the significant difference being observed till the end of the surgery. Moreover, the index returned to a normal level after the operation. Therefore, it was concluded that patients given MT showed more stable signs 15 minutes after the start of the operation, which greatly provides more evidence-based medicine for the application of MT.

## 6. Application of Music Therapy in Postoperative Nursing of General Surgery

### 6.1. Relief of Postoperative Anxiety

Postoperative anxiety is a common postoperative reaction of general surgery patients, in which the nervous and fearful emotions of the patient give rise to the excitement of the autonomic and sympathetic nerves, and the body is in a state of stress due to the surgical trauma. The human body under stress indicates the activation of the hypothalamic-pituitary-adrenal axis and the sympathetic nervous system, which leads to an increase in heart rate, blood pressure, and cardiac output, resulting in delays in recovery and discharge [23]. Traditional medicine only applies humanistic care and psychological intervention for adjuvant treatment of postoperative anxiety. The intervention method is relatively simple, and the effect requires further improvement. Based on the changes in hormone secretion in anxiety, Fu et al. [24] concluded that music can abate the neuroendocrine stress response caused by surgery. Kavak Akelma et al. [25] also stated that music can reduce anxiety, adjust hemodynamic parameters, and improve postoperative satisfaction. Sawni and Breuner [26] used MT and without MT (non-MT) for postoperative anxiety in 80 cases of liver cancer after Hepatic Artery Interventional Chemoembolization (TACE), evaluated their anxiety state through SAS, and found that the SAS score of patients in the non-MT group was much higher than that of the patients in the MT group. The SAS test was performed on the 4th day after surgery with the non-MT group of 45.2 ± 9.52 points and the MT group of 37.65 ± 8.54 points (*P* < 0.05), which proved the effectiveness of music therapy in relieving postoperative anxiety. Furthermore, MT is also considered a noninvasive, well-tolerated, cost-effective, nonpharmacological, and low-risk intervention [27].

### 6.2. Treatment of Postoperative Pain

Postoperative pain of general surgery is inevitable, given its complexity and large trauma. Pain can reflexively stimulate the sympathetic nerves and thalamus, promote the secretion of catecholamine hormones, and increase metabolism and oxygen consumption, resulting in a state of negative nitrogen balance of the body. Consequently, patients may be predisposed to fatigue and postoperative pain due to the lowered pain threshold of patients under anxiety, which enlarges the demand for opioids [28]. The traditional postoperative analgesia generally adopts opioid analgesics such as fentanyl which have strong side effects, such as dizziness, nausea, vomiting, even drug dependence, and drug resistance [29]. Music can prompt the reticuloendothelial system of the brainstem to slow down the input of painful stimulation, produce inhibitory nerve impulses, and shut down the conduction valve of painful nerve impulses, to eventually achieve pain relief. A meta-study by Poulsen and Coto [17] also pointed out that MT is 70% more effective for postoperative pain treatment than without MT, and that MT is effective during the medical process regardless of the time points of application. A meta-study by Kühlmann et al. [12] also concluded a reduction of the VAS pain score by at least 12 mm for postoperative pain after MT.

## 7. Issues in MT

With the continuous promotion of the concept of rapid rehabilitation surgery, the role of music therapy in the perioperative period continues to be explored in light of the conformity of MT with the modern biological-psychological-social medical model. Currently, it has now become a crucial part of the concept of rapid rehabilitation surgery with the continuous deepening of research. In the COVID-19 pandemic, MT was also applied in the Fangcang mobile hospitals, which reflects the popularity of MT.

The current issues with MT in China mainly include (1) the deficiency of professional music therapists. As MT started late in China, there is still a great need for professional music therapists in current clinical practice; (2) low acceptance of MT. Except for special medical institutions such as nursing homes, ordinary medical institutions do not have sufficient awareness of MT.

## 8. Conclusion

There is no doubt about the effectiveness of MT, which is effective in the mitigation of negative emotions, the relief of pain, and even the stabilization of vital signs. In China, due to the late start and novelty of MT, as it is seldom used in patients. Around the world, MT is still in the exploratory stage. It is worth noting that in addition to general surgery, MT is also widely used in different diseases, and the large space for development reveals its vigorous prospect in the future.

## References

[1] Li G., Li M., Zhang J. (2021). Mechanism and research progresses of personalized music in the treatment of tinnitus. *Lin Chuang er bi yan hou tou Jing wai ke za zhi= Journal of Clinical Otorhinolaryngology, Head, and Neck Surgery*.

[2] Mitchell M. L., Henderson A., Groves M., Dalton M., Nulty D. (2009). The objective structured clinical examination (OSCE): optimising its value in the undergraduate nursing curriculum. *Nurse Education Today*.

[3] Hole J., Hirsch M., Ball E., Meads C. (2015). Music as an aid for postoperative recovery in adults: a systematic review and meta-analysis. *Lancet*.

[4] Taylor D. B. (1981). Music in general hospital treatment from 1900 to 1950. *Journal of Music Therapy*.

[5] Bruscia K. E. (1998). Standards of integrity for qualitative music therapy research. *Journal of Music Therapy*.

[6] Matsota P., Christodoulopoulou T., Smyrnioti M. E. (2013). Music's use for anesthesia and analgesia. *Journal of Alternative and Complementary Medicine*.

[7] Schneider M. A. (2018). The effect of listening to music on postoperative pain in adult orthopedic patients. *American Holistic Nurses' Association*.

[8] Hartling L., Newton A. S., Liang Y. (2013). Music to reduce pain and distress in the pediatric emergency Department. *JAMA Pediatrics*.

[9] Li Y., Xing X., Shi X. (2020). The effectiveness of music therapy for patients with cancer: A systematic review and meta‐analysis. *Journal of Advanced Nursing*.

[10] Thaut M. H. (2015). Music as therapy in early history. *Progress in Brain Research*.

[11] Hughes J. R., Fino J. J. (2000). The Mozart effect: distinctive aspects of the music--a clue to brain coding?. *Clinical EEG (electroencephalography)*.

[12] Kühlmann A. Y. R., de Rooij A., Kroese L. F., van Dijk M., Hunink M. G. M., Jeekel J. (2018). Meta-analysis evaluating music interventions for anxiety and pain in surgery. *The British Journal of Surgery*.

[13] Ferreri L., Mas-Herrero E., Zatorre R. J. (2019). Dopamine modulates the reward experiences elicited by music. *Proceedings of the National Academy of Sciences of the United States of America*.

[14] Son H. K., So W. Y., Kim M. (2019). Effects of aromatherapy combined with music therapy on anxiety, stress, and fundamental nursing skills in nursing students: a randomized controlled trial. *International Journal of Environmental Research and Public Health*.

[15] Vila M. R., Todorovic M. S., Tang C. (2020). Cognitive flexibility and persistent post-surgical pain: the FLEXCAPP prospective observational study. *British Journal of Anaesthesia*.

[16] Kipnis G., Tabak N., Koton S. (2016). Background music playback in the preoperative setting: does it reduce the level of preoperative anxiety among candidates for elective surgery?. *Journal of PeriAnesthesia Nursing*.

[17] Poulsen M. J., Coto J. (2018). Nursing music protocol and postoperative pain. *Pain Management Nursing*.

[18] Nelson K., Adamek M., Kleiber C. (2017). Relaxation Training and Postoperative Music Therapy for Adolescents Undergoing Spinal Fusion Surgery. *Pain Management Nursing*.

[19] Kain Z. N., Wang S. M., Mayes L. C., Krivutza D. M., Teague B. A. (2001). Sensory stimuli and anxiety in children undergoing surgery: a randomized, controlled trial. *Anesthesia and Analgesia*.

[20] White J. M. (1999). Effects of relaxing music on cardiac autonomic balance and anxiety after acute myocardial infarction. *American Journal of Critical Care: An Official Publication, American Association of Critical-Care Nurses*.

[21] Jiménez-Jiménez M., García-Escalona A., Martín-López A., De Vera-Vera R., De Haro J. (2013). Intraoperative stress and anxiety reduction with music therapy: a controlled randomized clinical trial of efficacy and safety. *Journal of Vascular Nursing*.

[22] Bae I., Lim H. M., Hur M. H., Lee M. (2014). Intra-operative music listening for anxiety, the BIS index, and the vital signs of patients undergoing regional anesthesia. *Complementary Therapies in Medicine*.

[23] Bally K., Campbell D., Chesnick K., Tranmer J. E. (2003). Effects of patient-controlled music therapy during coronary angiography on procedural pain and anxiety distress syndrome. *Critical Care Nurse*.

[24] Fu V. X., Oomens P., Sneiders D. (2019). The Effect of Perioperative Music on the Stress Response to Surgery: A Meta- analysis. *The Journal of Surgical Research*.

[25] Kavak Akelma F., Altınsoy S., Arslan M. T., Ergil J. (2020). Effect of favorite music on postoperative anxiety and pain. *Der Anaesthesist*.

[26] Sawni A., Breuner C. C. (2017). Clinical hypnosis, an effective mind-body modality for adolescents with behavioral and physical complaints. *Children*.

[27] Uyar M., Akın Korhan E. (2011). The effect of music therapy on pain and anxiety in intensive care patients. *Aǧrı*.

[28] Palada V., Kaunisto M. A., Kalso E. (2018). Genetics and genomics in postoperative pain and analgesia. *Current Opinion in Anaesthesiology*.

[29] Volkow N. D., Jones E. B., Einstein E. B., Wargo E. M. (2019). Prevention and treatment of opioid misuse and addiction: a review,.

